# Statement on medication simplification in long‐term care facilities by the Japanese Society of Geriatric Pharmacy: English translation of the Japanese article

**DOI:** 10.1111/ggi.15009

**Published:** 2024-12-04

**Authors:** Hiroshi Maruoka, Shota Hamada, Eriko Koujiya, Kazumi Higashihara, Hiroshi Shinonaga, Katsuaki Arai, Saiko Saotome, Takashi Okura, Fumihiro Mizokami, Jiro Okochi, Yasushi Takeya, Naomi Kurata, Masahiro Akishita, Japanese Society of Geriatric Pharmacy Working Group on Medication Simplification in Long‐term Care Facilities

**Affiliations:** ^1^ Yokohama Aobanosato Geriatric Health Services Facility Kanagawa Japan; ^2^ Research Department Institute for Health Economics and Policy, Association for Health Economics Research and Social Insurance and Welfare Tokyo Japan; ^3^ Department of Geriatric Nursing, Division of Health Sciences, Graduate School of Medicine The University of Osaka Osaka Japan; ^4^ Baptist Geriatric Health Services Facility Kyoto Japan; ^5^ Department of Pharmacy, Mitoyo General Hospital Kagawa Japan; ^6^ Department of Pharmacy, Ooarai Seashore Hospital Ibaraki Japan; ^7^ Department of Pharmacy Iwaki Chuo Hospital Fukushima Japan; ^8^ Laboratory of Pharmaceutics, Faculty of Pharmaceutical Sciences Teikyo University Tokyo Japan; ^9^ Department of Pharmacy, National Center for Geriatrics and Gerontology Aichi Japan; ^10^ Tatsumanosato Geriatric Health Services Facility Osaka Japan; ^11^ Division of Clinical Nutrition and Metabolism, Department of Clinical Pharmacy, School of Pharmacy Showa University Tokyo Japan; ^12^ Division of Social Pharmacy, Department of Healthcare and Regulatory Sciences, School of Pharmacy Showa University Tokyo Japan; ^13^ Tokyo Metropolitan Institute for Geriatrics and Gerontology Tokyo Japan

**Keywords:** long‐term care facilities, medication regimen complexity, medication simplification, nursing homes, older adults

## Abstract

Many older adults who are certified for long‐term care services live or stay in long‐term care facilities (LTCFs), where they receive medical and nursing care. These individuals often encounter medication‐related problems, such as polypharmacy and complex medication regimens, including frequent administration schedules. Although considerable attention has been paid to polypharmacy in the context of optimizing medication use in older adults, little emphasis has been placed on simplifying these regimens. Recently, the Japanese Society of Geriatric Pharmacy issued statements on medication simplification in LTCFs based on a scoping review of the literature and expert opinions. In these statements, medication simplification is defined as the process of reducing the number of medication administration times, ideally to once during lunchtime. The statements outline principles and processes to achieve medication simplification through interprofessional collaboration, including consolidating and reducing the number of medication administration times a day to minimize the risk of medication errors and ensure medical safety. Medication simplification will play a substantial role in alleviating the burden of medication intake for residents, and in reducing and equalizing the workload of medication administration for staff members throughout the day in LTCFs. These statements suggest that administering medication during lunchtime is beneficial when an adequate number of staff members are available in LTCFs. We hope that these statements will help ensure patient safety, and facilitate successful medication optimization for all medical, nursing and social care professionals working in LTCFs. **Geriatr Gerontol Int 2025; 25: 14–24**.

## Introduction

As Japan faces a super‐aged society, there has been a marked increase in the number of older adults with multiple chronic diseases and conditions (i.e. multimorbidity) who require long‐term care. Many of these individuals live in long‐term care facilities (LTCFs), where they receive both medical and nursing care; however, they often face challenges with medication management. These challenges are attributed to various factors, including a decline in cognitive function, reduced motor function (such as manual dexterity) and swallowing difficulties. Additionally, polypharmacy and staff shortages within facilities providing medication support contribute to these challenges. Although facilities organize staff shifts to administer and monitor medications at various times (morning, noon, evening and before bedtime), current medications make it difficult to ensure proper medication support amid other care responsibilities.

First, it is necessary to conduct a medication review to address polypharmacy with the aim of reducing the number of medications to the minimum required. However, even with effective medication reviews, problems with medication management persist if multiple medications remain.

What can be done in such a case? One solution is to reduce the number of medication administration times, ideally just once daily. Considering the characteristics of LTCFs, it might be most effective to concentrate medication administration during lunchtime, when staffing levels are at their highest. This suggestion arose from medical professionals and nursing care staff working in LTCFs. The Japanese Society of Geriatric Pharmacy formed a working group in September 2023 to test this hypothesis. Although the potential benefits for both the facility and residents are substantial, challenges were also identified, such as the unsuitability of certain medications administered during lunchtime and drug–drug interactions. After carrying out a scoping review and other activities, the “Statement on medication simplification in long‐term care facilities” was finalized.^1^

The concept of medication simplification and medication administration during lunchtime applies not only to residents of LTCFs, but also to older adults in other settings, including during hospitalization and home medical care. Further consideration should be given to these aspects.

We hope all medical, nursing and social care professionals, including physicians, dentists, pharmacists and nurses, will use these statements.

## Statements on medication simplification in long‐term care facilities

Statement 1. Reducing the number of medication administration times offers multiple benefits.

Reducing the number of medication administration times can reduce the risk of medication errors and improve medical safety. For residents, this can reduce the burden of taking medications and improve adherence. Facility staff can reduce the workload related to medication administration and help balance work schedules.

Statement 2. Once a day during lunchtime: consider consolidating medication administration to once during lunchtime whenever feasible.

Consolidating medication administration during lunchtime, when staffing levels are high, can offer additional benefits. However, some medications might not be suitable for administration during lunchtime. Adjustments might be necessary if the care settings change.

## What is medication simplification?

**Figure 1:**
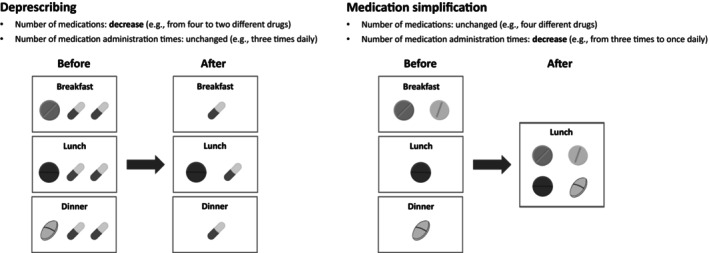
Contrast between deprescribing and medication simplification.

In these statements, medication simplification refers to the reduction of the number of medication administration times, ideally consolidating them preferentially into a single administration during lunchtime. Medication simplification is an inherent part of polypharmacy management; however, these statements focus specifically on medication simplification, independent of the reduction in the number of medications (i.e. deprescribing). The contrast between deprescribing and medication simplification is shown in Figure 1.

**Figure 2:**
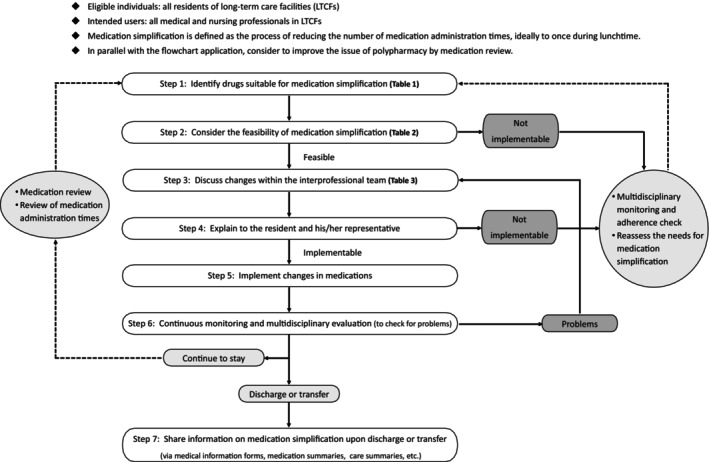
Flowchart for medication simplification in long‐term care facilities.

In LTCFs, it is advisable to actively pursue medication simplification along with medication reviews. As care needs and staff workload increase for older residents, excessive time and effort spent on medication management can lead to accidents or a decline in the quality of care. These statements provide guidelines for implementing medication simplification in LTCFs (see “Flowchart for medication simplification in long‐term care facilities”; Fig. 2), and outline an approach for interprofessional collaboration.

In polypharmacy management, even after a medication review and a reduction in the number of medications, the number of medication administration times might remain unchanged. By contrast, medication simplification focuses on reducing the number of medication administration times, even if the number of medications remains unchanged (from three times a day to once daily; Fig. 1).

## Significance of medication simplification

### Medications in LTCFs

Residents of LTCFs often face various health issues, including frailty and cognitive decline, and require nursing care. In pharmacotherapy, the aforementioned residents receive polypharmacy by individually treating multiple diseases and symptoms. Polypharmacy is defined not merely as the use of a large number of medications, but also as a state where the use of medications is associated with increased risks of adverse drug events, medication errors and reduced adherence. Polypharmacy is a critical issue in pharmacotherapy for residents of LTCFs. A medication review should be carried out to assess the necessity and prioritize medications, select only essential medications, and deprescribe when possible.^2^

Along with medication reviews, reviewing and reducing the number of medication administration times can lead to improved pharmacotherapy. Even if the number of medications remains unchanged, differences in dosing frequency and formulation can complicate medication regimens.^3^, ^4^ Increased complexity of medication regimens can elevate the risk of medication errors, increase the time and effort required for administration (especially when support is needed), and lead to reduced adherence, increased risks of hospitalizations, readmissions, emergency visits and even mortality.^4^, ^5^, ^6^, ^7^ An association between medication regimen complexity and the risk of hospitalization has been observed in residents of LTCFs.^8^ Therefore, incorporating the perspective of medication simplification, separate from deprescribing, into pharmacotherapy for residents of LTCFs is essential. Additionally, considering the possibility of medication management on their own in certain facilities or after residents return home, simplifying medication to make it easier to manage independently is crucial for maintaining good adherence.

Among the methods used to quantitatively evaluate the complexity of medication regimens, the Medication Regimen Complexity Index (MRCI) is the most widely used.^9^ The MRCI evaluates the complexity of medication regimens based on three sections: section A (dosage forms), section B (dosing frequency) and section C (additional administration directions), with a higher total score indicating greater complexity.^3^ Studies carried out in other countries have shown high medication regimen complexity among residents of LTCFs.^10^, ^11^, ^12^, ^13^, ^14^, ^15^ In recent years, some studies using MRCI have been published in Japan,^16^, ^17^, ^18^, ^19^ involving hospitalized patients and older adults receiving home medical care. MRCI scores from studies carried out in Japan were relatively lower than those reported in studies with residents of LTCFs carried out in other countries. Nevertheless, there is still potential for medication simplification among residents of LTCFs in Japan.

However, using the MRCI involves challenges, such as the time and effort required for scoring. Notably, section B (dosing frequency) contributes the most to the MRCI score,^10^, ^12^, ^13^, ^20^ and there is a correlation between the MRCI score and dosing frequency.^17^, ^21^ However, as the MRCI evaluates each medication individually, assessing the total number of administration times is also essential. A correlation has been observed between the number of administration times and the MRCI score, particularly with the dosing frequency.^13^ Studies performed outside of Japan have shown that pharmacist‐led interventions using a structured method (such as changing administration times or formulations) typically reduce medication administration times with a favorable safety profile.^22^, ^23^, ^24^

### Burden of medication administration on facility staff and medical safety

The complexity of medication regimens also poses problems when the burden of medication administration on nurses and nursing care staff is considered. In facilities, nurses are primarily responsible for daily medication management, and nursing care staff are involved in medication distribution. However, in facilities without resident physicians or pharmacists who have expertise in medications, the burden of medication management on nurses and nursing care staff is considerable.^25^, ^26^ In LTCFs in Japan, 40–50% of staff report feeling burdened by supporting medication administration.^25^, ^27^

Furthermore, approximately 60% of residents of LTCFs require support with medication administration. These residents are often prescribed multiple medications owing to multimorbidity, and the routes (e.g. oral, topical, injection [e.g. insulin]), timing (e.g. before or after meals) and frequency (e.g. biweekly) of administration vary. Adherence to the administration directions tailored to each resident is required. Additionally, medication administration support must be adjusted for the physical and cognitive decline that comes with aging, making the burden on caregivers considerable.^28^

Staff shortages in facilities also pose a critical issue in medication management. Although fewer staff are available at night compared with daytime, medication administration is often concentrated after breakfast or dinner, coinciding with night shifts.^29^ By concentrating medication administration during lunchtime, considering the allocation of nurses and nursing care staff and their working hours, the burden on caregivers can be reduced, allowing time for other tasks and potentially improving the overall quality of care. Some drugs are not suitable to take during the daytime. When these drugs are included in the medication, consider consolidating the timing of medication administrations as once in the morning, evening or before bed, or not consolidating the administration timing.

Furthermore, the burden on nurses and nursing care staff, as well as staff shortages, raise concerns about medical safety. In LTCFs in Japan, medication errors are frequently reported after incidents of falls and injuries.^30^ This issue is not unique to Japan; similar trends have been observed internationally.^31^, ^32^, ^33^, ^34^ Contributing factors might include staff shortages, interruptions in medication administration or distribution due to other tasks and the complexity of medication regimens. Medication simplification is necessary to prevent medication errors in distribution or administration and to ensure the safety of residents.

## Flowchart for medication simplification in LTCFs

The process of implementing medication simplification in LTCFs is outlined in the seven steps shown in Figure 2. For details on the points to consider during the implementation, please refer to the following section.

### Before implementing medication simplification

All medical professionals and nursing care staff in LTCFs are expected to use these statements. The target population includes all residents of LTCFs. To smoothly implement medication simplification, it is advisable to unify staff members' understanding on the concept of medication simplification, the process and points to consider, and each professional's roles through internal/external training and other means. Medication simplification should only be initiated when the resident's condition is stable, not during the acute phase. It is advisable to carry out medication reviews in parallel with the implementation of medication simplification.

### Step 1: Identify drugs suitable for medication simplification

Review all medications (including over‐the‐counter drugs) being used by residents, understand the frequency and timing of medication administration, and identify drugs that can be simplified. During this process, consider whether the simplification methods listed in Table 1 could be applied.

**Table 1:** Examples of medications suitable for simplification

Target	Medication simplification methods	Examples
Individual drugs	Adjusting the dosing frequency	Some medications that have adjustable dosing frequency in their package inserts can be consolidated from 2 to 3 times a day to once a day
	Switching to sustained‐release formulations	Some medications that are administered 2 to 3 times a day can be switched to sustained‐release formulations (including switching from oral medications to sustained‐release patches), thereby reducing the number of medication administration
Overall medication	Unifying the timing of medication administration	Some medications that are administered once daily can have their timing adjusted, allowing all doses to be administered at the same time, such as after lunch or before bedtime

### Step 2: Consider the feasibility of medication simplification

Check the identified drugs against the considerations listed in Table 2 to determine whether there are any challenges that would make medication simplification challenging for residents.

**Table 2:** Considerations for identifying medications for simplification

Does the usage or dosage deviate from the instructions in the package insert? Are there any safety concerns (e.g. side‐effects)? Will there be any increase or decrease in drug effectiveness (e.g. drug–drug interactions, food–drug interactions)? Will there be considerable inconvenience to the resident (e.g. difficulty swallowing tablets owing to the increased number of tablets, increased number of blood tests and increased costs)? Will there be considerable inconvenience to the facility (e.g. increased staff workload, increased costs)? Can the resident and his/her representative be adequately informed, and are they likely to provide their consent?

### Step 3: Discuss changes within the multidisciplinary team

When implementing medication simplification, refer to the main roles of each profession, as listed in Table 3, and discuss the feasibility and methods of medication simplification within the multidisciplinary team. Consider the residents' living conditions, medication adherence, potential for returning home, and the facility's staffing levels.

**Table 3:** Main roles by profession according to the flowchart for medication simplification in long‐term care facilities

Profession	Identification and evaluation of applicability of drugs for simplification (Steps 1–3)	Implementation and assessment after simplification (Steps 4–7)
Physician	Identify medications for simplification based on medical evaluation and assess feasibility (e.g. safety consideration, disadvantages to the resident or facility)	Implement changes in medications, explain to the resident and his/her representative, monitor and share the effectiveness and adverse drug events, and communicate concerns to the multidisciplinary team
Pharmacist	Identify medications for simplification based on pharmaceutical evaluation and assess feasibility (e.g. review package inserts and interview forms, consideration of interactions), propose changes in medications.	Provide explanations and supplement understanding to the resident and his/her representative, monitor and share the effectiveness and adverse drug events, integrate information from the multidisciplinary team, and communicate concerns to the multidisciplinary team
Nurse	Share information with the multidisciplinary team (medication adherence, medication management ability, status of medication support, ADL, living conditions, and burden and issues of medication management support), propose dosage forms, administration frequency, and administration methods that align with medication adherence and management abilities	Supplement understanding for the resident and his/her representative, monitor and share symptoms and adverse drug events due to the changes in medications, and adjust care according to changes in ADL and living conditions
Care worker Social worker Care manager, etc.	Share information with the multidisciplinary team (status of medication support, difficulty swallowing or medication refusal, medication adherence and living conditions before admission, burden and issues of medication administration support)	Monitor and share changes in medication adherence and living conditions due to the changes in medications, and provide medication‐related information to medical institutions and care service providers on discharge
Dentist Dental hygienist	Evaluate oral environment and swallowing function, propose dosage forms and administration methods suitable for oral intake	Monitor for impaired swallowing function as adverse drug events
Physical therapist Occupational therapist Speech therapist	Evaluate physical and swallowing function related to medication intake, propose dosage forms and administration methods suitable for oral intake	Monitor and share changes in physical and swallowing functions due to the changes in medications
Dietitian	Share information on appetite, preferences, food intake, food form and nutritional status.	Monitor and share changes in appetite, preferences, food intake, food form and nutritional status due to the changes in medications

ADL, activities of daily living.

### Step 4: Explain to the resident and his/her representative

Provide explanations to the resident and his/her representative. If medication simplification is difficult to implement, clarify the reasons, consider whether the issues can be resolved and, if possible, attempt it again.

### Step 5: Implement changes in medications

Implement changes in medications. Ensure that information is shared with the resident and his/her representative, and the multidisciplinary team.

### Step 6: Continuous monitoring and interprofessional evaluation

After implementing changes in medications, the residents' condition is continuously monitored, and effects of the change are evaluated by a multidisciplinary team. If problems arise, the team discusses ways to address them. If a resident continues to stay at the facility, continue to review the potential for further medication simplification.

### Step 7: Share information on medication simplification upon discharge or transfer

When residents are discharged, transferred, or admitted to another hospital or facility, share information regarding medication simplification through medical information sharing forms, medication summaries and nursing summaries.

## Points to consider during an intervention

This section provides points to consider when implementing the seven steps outlined in the previous section.

### Step 1: Identify drugs suitable for medication simplification

Several factors must be considered when identifying medications that can be simplified, as shown in Table 1.

When changing the dosing frequency from two or three times a day to once a day (Table 1), there are some drugs for which this change might not be appropriate. Some medications, such as anti‐Parkinson drugs, require divided dosing to be effective,^35^ and medication simplification can increase the risk of worsening symptoms.

When considering a switch to sustained‐release formulations, keep in mind that extending the half‐life of the drug might prolong the duration of adverse effects. Additionally, for residents who tend to chew tablets, the release mechanism of sustained‐release formulations might be compromised, leading to unexpected adverse effects owing to a rapid increase in blood concentration, or conversely, reduced effects. When switching to sustained‐release patches, skin reactions, such as redness or inflammation, are observed. When using drugs with long dosing intervals, such as weekly or monthly formulations, there is a risk of missed doses or administrations owing to irregular timing; therefore, it is necessary to establish clear medication management methods at each facility (such as standardizing the administration day of the week).

Unify medication administration timing (e.g. once daily during lunchtime), if possible, considering that some medications, due to their properties, are best taken before meals (such as certain diabetes medications) or between meals (such as medications affected by food intake). Similarly, some medications are best administered in the morning (e.g. SGLT2 inhibitors), whereas others are best administered before bedtime (e.g. hypnotics and certain antihistamines with sedative effects). Therefore, the unification of the timing of medication administration should be considered on a case‐by‐case basis.

Considering unit dose packaging as a means of enhancing safety management, reducing workload and improving medication adherence is an effective strategy. However, some medications are unsuitable for unit dose packaging (e.g. highly hygroscopic drugs or those that are unstable in light), and the associated costs and workload should also be considered.

### Step 2: Consider the feasibility of medication simplification

When evaluating the feasibility of medication simplification, as outlined in Table 2, consider the following points:

The timing of medication administration cannot be changed owing to the characteristics of the drugs, for which the usage and dosage are restricted by the drug's package insert (e.g. some osteoporosis drugs at waking up and some antidiabetic drugs before meals).

Simplifying medication regimens might lead to an increased risk of aspiration or drug–drug interactions due to the higher number of medications taken simultaneously.^36^ Additionally, changing the timing of medication administration can elevate the risk of adverse effects. When considering drug–drug interactions and side‐effects, it is necessary to account not only for the effects of meal and interactions between drugs (e.g. reduced effectiveness owing to reduced drug absorption), but also for the decreased drug metabolism in residents.^37^ As a preventive measure against aspiration, it is crucial to evaluate the patient's physical function, including swallowing ability, and select appropriate drug forms and sizes based on the individual's condition.^38^, ^39^, ^40^

Medication simplification should avoid disadvantaging residents and facilities. It is important to consider the potential increase in financial burden due to changes in the formulations. When implementing these measures, it is advisable to assess the likelihood of obtaining consent through explanations to the resident and his/her representative.

### Step 3: Discuss changes within the multidisciplinary team

When discussing changes in medication within the multidisciplinary team, as outlined in Table 3, it is important to flexibly determine each professional's role, considering staffing levels and working hours of the facility. Although physicians and pharmacists primarily handle the identification of medications suitable for simplification and the feasibility of implementation, it is essential to share the necessary information for medication simplification across the multidisciplinary team, including residents' conditions. Establishing information‐sharing methods among the professionals (e.g. regular conferences) in advance will facilitate smooth discussion.

### Step 4: Explain to the resident and his/her representative

When implementing medication simplification, explanations to the resident and his/her representative are crucial, because an inadequate understanding can lead to medication errors and refusal to take medications or mistrust of pharmacotherapy. A resident's understanding and trust in healthcare providers substantially affects medication adherence^41^; therefore, careful support from a multidisciplinary team is advisable. Medication simplification is also highly beneficial when aiming for returning home. It is necessary to carefully explain medication simplification to avoid the potential risks of medication errors or decreased adherence due to insufficient understanding by the resident or his/her representative.

### Step 5: Implement changes in medications

When implementing changes in medications, the timing of when to start the medication simplification should be clearly defined. Changes might take effect the following day or once the current medication supply is exhausted. Ensure that this information is reliably shared across the multidisciplinary team. In addition, reasonable measures should be taken regarding costs and workload, considering the facility's medication dispensing situation (e.g. outsourcing).

### Step 6: Continuous monitoring and interprofessional evaluation

After implementing medication simplification, it is essential to monitor residents' understanding of changes in medications and their adherence. Additionally, follow up on residents' health and regularly evaluate the impact of medication simplification. To achieve this, establishing interprofessional conferences or other means to periodically discuss and evaluate these outcomes is advisable. If interprofessional conferences are difficult to hold due to staffing levels and working hours, establishing methods for sharing information among professionals in advance (e.g. morning briefings, internal emails and bulletin board functions in medical records) can facilitate smooth follow up. If problems arise due to medication simplification, the multidisciplinary team should promptly address them. Regular discussions regarding further medication simplification are advisable, even if the patient's condition is stable.

### Step 7: Share information on medication simplification on discharge or transfer

Sharing information on the medication simplification implemented with medical institutions or facilities on discharge or transfer includes not only the medication information at the time of discharge or transfer, but also the details of the medication simplification process to ensure continuous care.

## Summary of literature review

To develop the statement, we carried out a scoping review^42^ to identify and organize the existing relevant knowledge. The research question was, “What are the current situation and impacts of medication regimen complexity in residents of LTCFs, and what are the interventions for medication simplification and their effects?” We carried out a literature search on 6 March 2024, using PubMed and Igaku Chuo Zasshi (ICHUSHI) databases. The search strategy for PubMed was “(medication* OR drug* OR prescri* OR polypharmacy OR pharmacotherapy) regimen* (complex* OR simpl*) (“long‐term care” OR “aged care” OR “nursing home*” OR “care home*” OR “group home*” OR institutionali*),” whereas for ICHUSHI, we used the Japanese terms “LTCFs AND drugs” in original research articles.

Overall, 141 and 891 articles were retrieved from PubMed and ICHUSHI, respectively. After screening the titles and abstracts, 16 articles (four studies carried out in Japan and 12 studies carried out in other countries) were selected for full‐text review and analysis. Based on the characteristics of the studies, we categorized the research into four groups: (A) the current situation of medication regimen complexity, (B) the impact of medication complexity, (C) interventions for medication simplification and their effects, and (D) the burden of medication support. A summary of each study in these categories is presented in Table 4. Categories A–C included only studies carried out overseas, whereas category D consisted only of studies carried out in Japan.

**Table 4:** Summary of literature analyzed

Study (first author, publication year)	Study methods	Medication regimen complexity and related main results
Current situation of medication regimen complexity		
Advinha, 2014^10^	Cross‐sectional study Portugal, five facilities Conducted in 2009 Residents (*n* = 415, mean age 83.9 years, female 60.2%)	Mean number of drugs: 8.2 Mean MRCI score: total, 18.2; section A, 3.6; section B, 11.2; section C, 3.4 Common dosing frequency: twice daily (31.8%)
Herson, 2015^11^	Cross‐sectional study Australia, six facilities Conducted in 2014 Residents (*n* = 383, mean age 87.5 years, female 77.5%)	Median number of drugs: 13 (including as‐needed drugs) Median MRCI score: total, 43.5 (no data by sections reported) Common dosing frequency: once daily (54.6%)
Alves‐Conceição, 2017^12^	Cross‐sectional study Brazil, three facilities Conducted in 2015 Residents (*n* = 125, mean age 81.8 years, female 64.4%)	Mean number of drugs: 4.0 Mean MRCI score: total, 15.1; section A, 4.6; section B, 5.5; section C, 4.9
Chen, 2019^13^	Cross‐sectional study Australia, eight facilities Conducted in 2017 Residents (*n* = 242, median age 87 years, female 74%)	Median number of drugs: 9 Median MRCI score: total, 42; section A, 9; section B, 19.8; section C, 11 Common dosing frequency: once daily (63%; regular drugs) Positive correlation of medication administration times and MRCI score (particularly section B)
Page, 2023^14^	Cross‐sectional study Australia, 17 facilities Conducted between 2014 and 2018 Residents (*n* = 303, mean age 85 years, female 76%)	Mean number of drugs (drug products): 9.5 Mean MRCI score: total, 43 (no data by sections reported)
Page, 2024^15^	Cohort study New Zealand, 34 facilities Conducted between 2017 and 2021 Residents at admission (*n* = 3802, mean age 84.9 years, female 61.2%) Residents 30 days later, *n* = 3305; 12 months later, *n* = 2140	Mean number of drugs (drug ingredients): 6.0 at admission, 6.6 at day 7, 7.0 at day 30 and 8.3 at month 12 Mean MRCI score: total, 33.4 at admission; 38.8 at day 7; 41.5 at day 30; 55.7 at month 12 (no data by sections reported)
Impact of medication complexity		
Lalic, 2016a^43^	Cross‐sectional study Australia, six facilities Conducted in 2014 Residents (*n* = 383, mean age 88 years, female 78%)	Median number of drugs: 10 Median MRCI score: 43.5 (no data by sections reported) Common dosing frequency: once daily 54.6% No associations between medication regimen complexity and health‐related quality of life
Lalic, 2016b^8^	Cohort study Australia, six facilities Conducted in 2014 Residents (*n* = 383, mean age 88 years, female 78%)	Median number of drugs: 10 Median MRCI score: 43.5 (no data by sections reported) Common dosing frequency: once daily 54.6% Medication regimen complexity was associated with a greater risk for hospitalization from LTCFs
Interventions for medication simplification and their effects		
Pouranayatihosseinabad, 2018^20^	Cohort study Australia, LTCFs and RMMR provider Conducted between 2011 and 2012 Residents (*n* = 285, mean age 85.6 years, female 67.7%)	Mean number of drugs: 8.8 (including as‐needed drugs) Median MRCI score (before RMMR): total, 25.5; section A, 5.0; section B, 12.0; section C, 8.0 Common dosing frequency: once daily 53.8% No significant decrease in medication regimen complexity by the RMMR
Sluggett, 2020a^22^	Cluster randomized controlled trial Australia, eight facilities Conducted in 2017 Intervention: once application of a structured tool for medication simplification Residents (*n* = 242, mean age 86 years, female 74%)	Median number of drugs: 12–13 (including as‐needed drugs) Mean medication administration times (at admission to 4 months later): intervention group 3.9–3.6 times, control group 4.0–4.0 times Recommendations in two‐thirds of residents; mean 1.48 recommendations; change in dosing time 65%, change of formulation 27%, change of dosing frequency 4%; implementation rate 62% No effects on falls, hospitalization and mortality
Sluggett, 2020b^23^	Cluster randomized controlled trial Australia, eight facilities Conducted in 2017 Intervention: once application of a structured tool for medication simplification Residents (*n* = 242, mean age 86 years, female 74%)	Median number of drugs: 12–13 (including as‐needed drugs) Mean number of medication administration times (at admission and month 4, 8, and 12): interventional group, 3.9, 3.6, 3.7 and 3.6 times, respectively; control group 4.0, 4.0, 4.1 and 4.1 times, respectively Increase in falls (between 4 and 8 months after admission in one facility) No effects on hospitalization and mortality
Dugré, 2021^24^	Cluster randomized controlled trial Australia, eight facilities Conducted in 2017 Intervention: once application of a structured tool for medication simplification Residents (*n* = 242, mean age 86 years, female 74%)	Median number of drugs: 12–13 (including as‐needed drugs) Median number of medication administration times: 4 times for both groups Decrease in incidents observed in both groups, but no differences between the groups
Burden of medication support		
Imadu, 2008^44^	Before and after study Special nursing home, one facility Conducted in 2006 Nurses Residents (*n* = 23)	Reduce the time for enteral nutrition management by changing the medication administration times from three times a day to twice a day
Maki, 2020^25^	Cross‐sectional study Residential facilities with long‐term care: 89 facilities Conducted in 2014 Nurses (*n* = 233) Nursing care staff (*n* = 1484)	Overall, 42.5% of staff felt medication support was burdensome (e.g. refusal to take medication, staff shortages, overlap with other tasks, be rushed, large numbers of drugs and medication administration times)
Akashita, 2021^29^	Cross‐sectional study LTCF, one facility Conducted in 2018 Nursing care staff (*n* = 20) Residents (*n* = 51)	The number of drugs to be administered was high, but the number of staff for medication administration was low in the morning Changing the timing of medication administration from once in the morning to once during lunchtime may reduce the medication administration tasks
Nagata, 2023^27^	Cross‐sectional study LTCFs, 60 facilities Conducted in 2020 Staff (*n* = 263)	Overall, 40–50% of staff felt medication support was burdensome, and this is possibly a reason for burnout

LTCF, long‐term care facilities; MRCI, medication regimen complexity index; RMMR, residential medication management review.

The main findings obtained are as follows: (i) medication complexity is relatively high in LTCFs; (ii) when evaluated using the MRCI, dosing frequency contributes most substantially to medication complexity; (iii) medication complexity in LTCFs is associated with an increased risk of hospitalization; and (iv) pharmacist‐led interventions can reduce the number of medication administration times and the burden of medication support without substantially increasing the occurrence of adverse events. However, there is insufficient evidence from relevant studies, and evidence from Japan is particularly scarce, indicating a need for further verification. Given the potential differences in the types of LTCFs, self‐management of medication or medication support, and staff allocation, it is necessary to establish medication simplification methods suitable for each facility.

## Disclosure statement

The authors declare no conflict of interest. Unrelated to this work, Shota Hamada belongs to an endowed chair funded by donations from Hakue technology, PROUMED, Japan Bio Products, Towa Pharmaceutical, Yellow Eight and Sugi Holdings; Naomi Kurata received payment from Daiichi Sankyo; and Masahiro Akishita received grants or contracts from Eisai, Kracie Pharma, Mitsubishi‐Tanabe Pharma and Tsumura, and received payment from Bayer HealthCare, Daiichi Sankyo, Toa Eiyo and Towa Pharmaceutical.

## Author contributions

Hiroshi Maruoka and Masahiro Akishita conceptualized the study with support from Shota Hamada, Eriko Koujiya, Kazumi Higashihara and Hiroshi Shinonaga. Hiroshi Maruoka, Shota Hamada, Eriko Koujiya, Kazumi Higashihara, Hiroshi Shinonaga, Katsuaki Arai and Saiko Saotome carried out the scoping review. Hiroshi Maruoka, Shota Hamada, Eriko Koujiya, Kazumi Higashihara and Hiroshi Shinonaga drafted the original manuscript, and the other authors revised it critically for important intellectual content. All authors approved the final version to be published.

## Funding statement

This research was funded by Japanese Society of Geriatric Pharmacy.

## Data Availability

Data sharing is not applicable to this article as no new data were created or analyzed in this study.
